# Lenalidomide versus lenalidomide + dexamethasone prolonged treatment after second‐line lenalidomide + dexamethasone induction in multiple myeloma

**DOI:** 10.1002/cam4.1422

**Published:** 2018-04-19

**Authors:** Johan Lund, Astrid Gruber, Birgitta Lauri, Adil Doganay Duru, Cecilie Blimark, Agneta Swedin, Markus Hansson, Karin Forsberg, Lucia Ahlberg, Conny Carlsson, Anders Waage, Peter Gimsing, Annette Juul Vangsted, Ulf Frølund, Erik Holmberg, Gösta Gahrton, Evren Alici, Mats Hardling, Ulf‐Henrik Mellqvist, Hareth Nahi

**Affiliations:** ^1^ Department of Hematology Karolinska University Hospital Stockholm Sweden; ^2^ Department of Internal Medicine Sunderby Hospital Luleå Sweden; ^3^ Nova Southeastern University (NSU) Fort Lauderdale Florida; ^4^ Department of Hematology Sahlgrenska University Hospital Gothenburg Sweden; ^5^ Department of Hematology Skåne University Hospital Lund Sweden; ^6^ Department of Hematology Norrland University Hospital Umeå Sweden; ^7^ Department of Hematology University Hospital of Linköping Linköping Sweden; ^8^ Department of Internal Medicine Hallands Hospital Halmstad Sweden; ^9^ Department of Cancer Research and Molecular Medicine Norwegian University of Science and Technology Trondheim Norway; ^10^ Department of Hematology Rigshospitalet Copenhagen Denmark; ^11^ Department of Hematology Zealand University Roskilde Denmark; ^12^ Department of Oncology Institute of Clinical Sciences Gothenburg Sweden; ^13^ Department of Hematology Uddevalla Hospital Uddevalla Sweden

**Keywords:** Clinical Trial, Lenalidomide, Multiple Myeloma

## Abstract

Lenalidomide (Len) plus dexamethasone (Dex) is approved for the treatment of relapsed or refractory multiple myeloma (RRMM). It is possible that single‐agent Len may be effective as prolonged treatment regimen in RRMM once patients demonstrate an initial response to Len+Dex induction. Patients with RRMM who responded to first‐line Len+Dex in an observational study (NCT01430546) received up to 24 cycles of either Len (25 mg/day) or Len+Dex (25 mg/day and 40 mg/week) as prolonged treatment in a subsequent phase 2 clinical trial (NCT01450215). In the observational study (*N* = 133), median time to response was 1.7 (range 0.6–9.6) months. A complete response to all treatments received in both studies was observed in 11% of patients; very good partial response and partial response rates were 31% and 38%, respectively. Corresponding response rates in the subgroup of patients who did not enter the phase 2 trial (*n* = 71) were 3%, 18%, and 39%, respectively. Rates of disease progression at 2 years in the phase 2 trial were 47% versus 31% for Len versus Len+Dex (*P* = 0.14). After 36 months median follow‐up in surviving patients, median time to progression was not reached with Len+Dex and was 24.9 months (95% confidence interval 12.5–not calculable, *P* < 0.001) with Len. Three‐year OS among the total observational study population was 61% (95% CI, 52–69%). The corresponding rate among patients who entered the phase 2 clinical trial was 73% (95% CI, 60–83%) and was significantly lower among those patients who achieved ≥PR but did not proceed into the phase 2 trial (55%; *P* = 0.01). In the phase 2 trial, OS was 73% in both treatment arms (*P* = 0.70). Neutropenia and thrombocytopenia were more common with prolonged (phase 2 trial) versus short‐term (observational study) Len administration but remained manageable. Prolonged treatment with Len with or without Dex provides sustained, clinically relevant responses and demonstrates an acceptable safety profile.

## Research in context

### Evidence before these studies

Therapeutic advances in recent years have significantly prolonged survival in patients with multiple myeloma (MM); nevertheless, almost all patients eventually relapse following first‐line treatment. In Europe, combination treatment with the immunomodulatory drug lenalidomide (Len) plus the glucocorticoid dexamethasone (Dex) is the standard of care for patients experiencing a first relapse. However, no studies published before the present research were undertaken reported rates of response to this regimen in patients who had received only one prior line of therapy.

Lenalidomide exerts its antimyeloma effects via a two‐pronged mechanism of action: It directly induces tumor cell death, while also stimulating the immune response to prevent disease recurrence. In vitro data demonstrate that although dexamethasone enhances the antiproliferative effects of lenalidomide when administered concomitantly, it simultaneously inhibits the drug's immunomodulatory properties in a dose‐dependent manner. It is possible that once patients achieve an initial response to treatment and the need for tumoricidal efficacy decreases, dexamethasone could be omitted from the treatment regimen to minimize glucocorticoid‐induced immunosuppression, and long‐term treatment continued with single‐agent lenalidomide. Studies have confirmed the efficacy of single‐agent lenalidomide as maintenance therapy in newly diagnosed myeloma, but data in the relapsed or refractory setting were previously lacking.

### Added value of these studies

The findings of our observational study confirm the value of Len+Dex as a first‐line treatment for RRMM, providing further evidence of the efficacy of this combination when administered to patients with MM at first relapse. The phase 2 trial results demonstrate, for the first time, that single‐agent Len yields similar survival benefits to Len+Dex when used in the prolonged setting in RRMM; they also add to the growing body of evidence indicating that continuous Len+Dex improves outcomes versus fixed‐duration therapy.

### Implications of all the available evidence

Our findings, taken in combination with those in previously published reports, support the positioning of Len+Dex as a first‐line treatment for patients with RRMM. Single‐agent Len is already established as prolonged treatment in newly diagnosed MM; we now provide evidence to suggest that prolonged treatment with single‐agent Len may also be an option for patients with RRMM, potentially providing similar survival benefits to those observed with Len+Dex.

## Introduction

The introduction of proteasome inhibitors (PIs) and immunomodulatory drugs has significantly prolonged survival for patients with multiple myeloma (MM) over the past 15 years 1, 2. The immunomodulatory drug lenalidomide (Len) has an established efficacy and safety profile in the treatment of MM 3, 4 and is approved in this indication in combination with dexamethasone (Dex) 5. The approval of Len for the treatment of relapsed or refractory MM (RRMM) was largely based on the results of two phase 3 trials 6, 7. A preplanned subset analysis of data from these trials found that Len+Dex administered at first relapse significantly prolonged median time to progression (TTP) in patients who had received only 1 versus ≥2 prior therapies (17.1 months vs. 10.6 months) 8. The complete response (CR) plus very good partial response (VGPR) rate was also significantly higher in patients experiencing first versus later relapse (39.8% vs. 27.7%). These findings suggest that Len+Dex is most beneficial in RRMM when administered early.

It is possible that once patients have achieved a response with Len+Dex, the need for tumoricidal effects subsides and the immunostimulatory effect of Len becomes of greater importance for the control of myeloma cells and, thus, disease progression. Potentially, Dex could be reduced or omitted to minimize glucocorticoid‐induced immunosuppression. A meta‐analysis of the results of three randomized, placebo‐controlled trials confirmed that single‐agent Len, administered as maintenance therapy in newly diagnosed MM following induction and consolidation treatment, *prolonged both progression‐free survival (PFS) and overall survival (OS) versus placebo* 9, 10, 11, 12. We therefore compared single‐agent Len versus Len+Dex as prolonged therapy for patients with RRMM who achieved at least a partial response (PR) following initial Len+Dex treatment at first relapse.

## Methods

### Study design and participants

Two studies were conducted in Denmark, Norway, and Sweden by the Nordic Myeloma Study Group. The study protocols were approved by the local ethics committee in Stockholm, Sweden. All patients provided written informed consent, and all procedures were conducted in accordance with the Declaration of Helsinki, the International Conference on Harmonization, and Guidelines for Good Clinical Practice.

#### Observational study

The first study was a multicentre, observational, noninterventional study (ClinicalTrials.gov registration number NCT01430546; protocol available online) in Len‐naïve patients with RRMM who were experiencing a first relapse. Relapse was defined as chemical relapse with no demands on CRAB criteria. Inclusion criteria included age ≥18 years and a diagnosis of MM confirmed by serum M‐protein >0.5 g/dL or urinary Bence Jones protein >200 mg/24 h. Patients with plasma cell leukemia, nonhematologic malignancies (except cancers of the skin, thyroid, cervix, breast, or prostate [Gleason grade ≤6], and cancers considered to be cured), or amyloidosis were excluded. Patients received Len 25 mg orally on days 1–21, and Dex 40 mg orally on days 1, 8, 15, and 22, of each 28‐day cycle. Each patient completed ≤9 treatment cycles, with clinic visits scheduled at the beginning of each cycle. Data were collected at each visit via questionnaires about symptoms and treatment side effects. All serious adverse events (SAEs) occurring from consent form signature until 30 days after the last dose of study drug or initiation of new anticancer therapy (whichever occurred first) were reported. Adverse events (AEs) and SAEs were documented using Medical Dictionary for Regulatory Activities (MedDRA) classification. Health‐related quality of life (HRQoL) was measured before the start of treatment, at 3 and 6 months, and 1 month after the end of treatment. Patients achieving ≥PR received two further cycles of Len+Dex consolidation with unmodified dose and were invited to join a prospective, randomized, open‐label, multicentre, phase 2 clinical trial (ClinicalTrials.gov registration number NCT01450215; protocol available online). No time elapsed from completing the phase 4 trial to enrollment in the phase 2 trial; all patients were on continuous treatment. Reasons for patients participated in the phase 4 trial to not take part in the randomized phase 2 trial were death (*n* = 3), adverse events (*n* = 20), progressive disease (*n* = 21), no response (*n* = 11), withdrawn consent (*n* = 1), problems with compliance (*n* = 1), and withdrawn consent (*n* = 1). The rest (*n* = 13) completed the nine planned cycles and did not want to take part in the randomized study. The median number of cycles in phase 4 study was four cycles (0.3–28).

Among patients who achieved ≥PR but did not participate in the phase 2 trial, reasons for nonparticipation were as follows: patient refusal to participate; AEs resulting in study drug dose modification or discontinuation; and disease progression before being invited to participate in the phase 2 trial. Observational study participants who did not enter the phase 2 trial received ≤9 cycles of Len+Dex and no further treatment thereafter.

#### Phase 2 clinical trial

##### Randomization

Phase 2 trial participants were randomized (fixed block 1:1) to prolonged treatment with either single‐agent Len or combined Len+Dex. Randomization was performed at the Hematology Center at Karolinska University Hospital, Sweden, using a central computer‐generated, Microsoft Excel‐based randomization system designed by the study statistician. When the study nurse entered information from a patient's consent form into the system, the program randomly allocated the patient to a treatment arm.

##### Procedures

Patients received either Len or Len+Dex for ≤24 cycles of 28 days’ duration. Patients in both treatment groups received Len 25 mg/day orally on days 1–21 of each cycle. Len+Dex patients also received Dex 40 mg orally on days 1, 8, 15, and 22 of each cycle. Clinic visits were scheduled at the beginning of each cycle. Data were collected at each visit via questionnaires about symptoms and treatment side effects. Depths of responses (PR, VGPR, or CR) were evaluated in comparison with M‐protein levels prior to entry in the observational study. All SAEs occurring from consent form signature until 30 days after the last dose of study drug or initiation of new anticancer therapy (whichever occurred first) were reported. After completion of 24 cycles, no further treatment was planned.

### Analysis of chromosomal abnormalities by fluorescent in situ hybridization

Chromosomal abnormalities were analyzed by fluorescence in situ hybridization (FISH). Briefly, CD138^+^ plasma cells (2–4 × 10^4^ cells/spot) were centrifuged to prepare hybridization slides using Cytospin (Thermo Scientific, Pittsburgh, PA), air‐dried overnight, and stored at −20°C. For FISH analysis, all cases were investigated with in vitro diagnosis‐certified probe sets targeting 1q21, del 17p13, del 13q14, and t(4;14) (Kreatech, Amsterdam, the Netherlands). Hybridization and signal detection were performed according to the manufacturer's protocols. Spot counting analysis was performed with an Olympus microscope (BX60, Tokyo, Japan). For each probe, 200 nuclei were evaluated. Frequencies of 20% (17p deletion) or 10% (all others) of cells were considered positive. Patients were categorized as high risk if they were positive for del 17p13, add 1q21, and/or t(4;14).

### EORTC quality of life questionnaire core module and multiple myeloma module

The European Organisation for Research and Treatment of Cancer (EORTC) quality of life questionnaire core module QLQ‐C30 is a 30‐item questionnaire that comprises nine multi‐item scales measuring various dimensions of HRQoL in patients with cancer 13. The EORTC multiple myeloma module QLQ‐MY20 is a 20‐item module that assesses symptoms/side effects and HRQoL issues specific to MM, and was designed for completion alongside the QLQ‐C30 14.

Both of these questionnaires were completed by observational study participants at baseline (before the start of study medication), after 3 and 6 months on treatment, and at the end of the study (1 month after the ninth treatment cycle). Patients completed the questionnaires unsupervised, at home. All responses were scored according to the developers’ instructions.

### Analysis of cell surface phenotype of T cells and natural killer cells by flow cytometry (phase 2 trial only)

For phenotypic characterization of T cells and natural killer (NK) cells, peripheral blood mononuclear cell (PBMC) samples were obtained at randomization and at three randomly selected timepoints during treatment. The samples were analyzed by flow cytometry with panels including fluorochrome‐conjugated monoclonal antibodies against the surface antigens, as described previously 15. Briefly, all vitally frozen patient PBMCs were thawed, washed with cold fetal bovine serum (FBS), and resuspended in cold phosphate‐buffered saline (PBS) supplemented with 2% FBS and 1 mmol/L ethylenediaminetetraacetic acid (EDTA). Antibody staining was performed as follows: Cells were washed once with PBS (containing 2% FBS and 1 mmol/L ethylenediaminetetraacetic acid [EDTA]) and incubated with antibody mixes at 4°C for 30 min. The labeled cells were then washed twice with PBS. Data acquisition was performed using a LSR II flow cytometer (Becton, Dickinson and Company) equipped with a high‐throughput system. The data were analyzed by FACSDiva (BD Biosciences, San Jose, CA) and FlowJo X software (TreeStar Inc., Ashland OR). The CD3^+^CD14^−^CD19^−^CD56^−^ population was used to identify T cells, while CD56^+^CD3^−^CD14‐CD19^−^ cells were defined as NK cells. Additionally, T cells were further subgrouped through CD45RA/CD45RO expression to identify naïve/effector T cells and memory T cells, respectively. Graphs were generated, and statistical analyses were performed by GraphPad Prism (GraphPad Software Inc. La Jolla, CA) and FlowJo X software (TreeStar Inc.).

### Statistical analyses

#### Observational study

Observational study outcome parameters were defined as follows: OS, time from study inclusion at first relapse until death from any cause; PFS, time from inclusion until disease progression or death; TTP, time from inclusion until objective tumor progression. The primary endpoints were time to best response (TTR), PR, VGPR, and CR.

#### Phase 2 clinical trial

Phase 2 trial outcome parameters were defined as follows: OS, time from randomization until death from any cause; PFS, time from randomization until disease progression or death; and TTP, time from randomization until objective tumor progression. The primary objective was to compare the efficacy of prolonged treatment with Len versus Len+Dex, where efficacy was measured by median TTP within a 24‐month timeframe. It was assumed that the mean difference in TTP between the two treatment groups should be zero, allowing for a lower equivalence bound of −8 months and an upper equivalence bound of 8 months, with an expected median TTP in the Len+Dex group of 18 months. For a significance level of 5%, a standard deviation of 10, and a power of 80%, it was calculated that 30 patients per treatment group would be sufficient (SAS version 9.2).

Statistical analyses were performed on the intention‐to‐treat population. For the phase 2 trial primary analysis, TTP was compared with Len versus Len+Dex using Kaplan–Meier methodology and the log‐rank test. The same methods were used for between‐treatment PFS comparisons. Proportions of patients were compared using the chi‐square test or, for low frequencies, Fisher's exact test. Continuous variables were compared using Student's *t*‐test.

Quantification of the burden of prolonged treatment with Len versus Len+Dex was a secondary objective of the phase 2 trial. Between‐group comparisons of the proportion of patients reporting AEs were performed by chi‐square test or, for low frequencies, Fisher's exact test. Phase 2 trial AEs were “new‐onset” AEs occurring after randomization. The safety analysis included data from all patients who received any study medication. The EORTC questionnaire results were compared at different timepoints using the chi‐square test. For NK/T cell evaluations (phase 2 trial), samples obtained during treatment were compared with those obtained at randomization using the unpaired *t*‐test.

The data analyses reported in this manuscript were performed by J.L., E.H., and H.N. All authors had access to the primary clinical trial data.

### Role of the funding sources

Both studies reported in this manuscript were funded by research grants from Celgene Corporation and the Swedish Cancer Society. Celgene Corporation also paid for the services of professional medical writers, who provided editorial assistance during the development of the manuscript. Neither of the funding sources contributed to the study design, nor to the collection, analysis, or interpretation of data.

## Results

### Patients and treatment

Between December 2010 and November 2013, 133 patients were enrolled into the observational study (Fig. S1). Sixty‐two of these patients subsequently entered the phase 2 trial and were randomized between June 2011 and February 2014. At the cut‐off date of March 31, 2016, the median (range) duration of follow‐up of all 133 patients was 3.3 (1.6–5) years. Baseline characteristics and first‐line treatments are summarized in Table 1. All patients had received only one prior line of treatment; 57% and 65% of patients in the observational study and phase 2 trial, respectively, had received high‐dose chemotherapy plus autologous stem cell transplantation (ASCT), and 73% and 64%, respectively, had been exposed to bortezomib and/or thalidomide. As shown in Table 1, the majority of patients had International Staging System (ISS) stage I or II disease at diagnosis; at randomization for the phase 2 trial, all patients had either stage I (63%) or stage II (38%) disease (data not shown). No patient had baseline creatinine clearance (CrCl) <15 mL/min, although baseline CrCl was <30 mL/min in two patients. In accordance with the product label, the Len dosage was reduced from 25 to 5 mg/day in these two patients, neither of whom participated in the phase 2 trial. Three patients enrolled in the phase 2 trial had dose reduction of Len to 15 mg in the phase 4 trial. Two of them continued on that dose in the phase 2 trial, and one had the dose restored to 25 mg at the time of randomization. Median follow‐up in both the observational study and the phase 2 trial was 3 years (3.4 years for surviving patients).

**Table 1:** Baseline characteristics and the first‐line treatment in the overall population included in the observational study and in the subpopulation of patients who proceeded into the phase 2 trial

	Observational study (*N* = 133)	Phase 2 trial Len (*n* = 31)	Phase 2 trial Len+Dex (*n* = 31)	*P*
Median (range) age, years	67.5 (34.9–85.9)	65.0 (47.8–78.6)	67.2 (44.9–82.0)	0.284
≥75 year, *n* (%)	30 (23)	4 (13)	6 (19)	
<75 year, *n* (%)	103 (77)	27 (87)	25 (81)	
Female/male sex, %	63/70 (47)	16/15 (52)	17/14 (55)	0.803
ECOG score, *n* (%)				
0	56 (43)	14 (45)	15 (48)	0.994
1	53 (41)	12 (39)	13 (42)	
2	14 (11)	3 (10)	–	
3	2 (2)	–	–	
Unknown	5 (4)	2 (6)	3 (10)	
ISS disease stage at diagnosis, *n* (%)				
I	29 (22)	6 (19)	8 (26)	
II	52 (39)	16 (52)	11 (35)	
III	15 (11)	1 (3)	4 (13)	
Unknown	37(28)	8 (26)	8 (26)	
M‐component, *n* (%)				
IgA	27 (20)	0 5 (16)	0 7 (23)	0.721
IgG	88 (66)	23 (74)	19 (61)	
IgM	1 (1)	0 (0)	0 (0)	
Bence Jones	6 (8)	0 3 (10)	2 (6)	
Unknown	11 (8)	0 (0)	0 3 (10)	
Light chain type, *n* (%)				
Kappa	83 (62)	21 (68)	21 (68)	0.852
Lambda	44 (33)	10 (32)	9 (29)	
Unknown	6 (5)	1 (2)	1 (3)	
Hemoglobin, g/L				
Mean (SD)	115 (17)	116 (15)	118 (12)	0.681
Median (range)	115 (63–155)	118 (90–143)	119 (84–138)	
Not available	3	0	0	
Creatinine, μmol/L				
Mean (SD)	82 (31)	78 (20)	72 (23)	0.361
Median (range)	75 (42–270)	75 (33–136)	68 (47–164)	
Not available	3	0	0	
Albumin, g/L				
Mean (SD)	35.3 (4.4)	34.8 (6.9)	35.3 (3.1)	0.734
Median (range)	36 (25–44)	36 (26–43)	35 (29–41)	
Not available	3	0	0	
β_2_ microglobulin, mg/L				
Mean (SD)	3.5 (1.6)	2.3 (1.0)	2.3 (1.0)	0.484
Median (range)	3.3 (1.5–7.7)	2.1 (1.6–5.3)	2.1 (1.0–5.2)	
Not available	96	11	11	
Calcium, mmol/L				
Mean (SD)	2.34 (0.2)	2.18 (0.3)	2.15 (0.4)	0.774
Median (min–max)	2.30 (1.90–3.98)	2.20 (2.04–2.68)	2.10 (2.04–2.70)	
Not available	27	0	0	
Bone disease, *n* (%)				
No	24 (18)	3 (10)	6 (19)	0.322
Yes	101 (76)	27 (83)	23 (75)	
Unknown	8 (6)	1 (3)	2 (6)	
Prior ASCT, *n* (%)	76 (57)	20 (65)	20 (65)	0.739
Prior PI and/or immunomodulatory drug, *n* (%)				
Bortezomib	76 (57)	18 (58)	12 (39)	0.177
Thalidomide	13 (10)	1 (3)	4 (13)	
Bortezomib + thalidomide	8 (6)	1 (3)	2 (6)	
Other	33 (25)	10 (33)	13 (42)	
Unknown	3 (2)	1 (3)	0 (0)	

ASCT, autologous stem cell transplantation; ECOG indicates Eastern Cooperative Oncology Group; ISS, International Staging System; PI, proteasome inhibitor; SD, standard deviation.

In the observational study, median treatment duration from inclusion (excluding phase 2 trial treatment) was 3.7 (range 0.2–9) months. Fifty‐seven percent of observational study participants completed the planned number of treatment cycles; reasons for premature discontinuation were as follows: disease progression (16%); AEs or SAEs (16%); no response (8%); poor treatment adherence (2%); and withdrawal of consent (1%). Median treatment duration from randomization in the phase 2 trial was 12.2 (range 0.7–48) months, with 35% of patients completing the maximum 24 treatment cycles. Reasons for premature discontinuation in the phase 2 trial were as follows: AEs/SAEs (27%); disease progression (34%); withdrawal of consent (3%); and poor treatment adherence (1%).

### Efficacy

#### Observational study

Median TTR and time to best response in the observational study (*N* = 133) were 1.7 (range 0.6–9.6) and 2.5 (range 0.6–28) months. Rates of CR, VGPR, and PR following all treatments received in both this study and the phase 2 trial are summarized in Table 2. The median duration of response (DoR) among the total observational study population was 18.2 (range 1.6–57) months.

**Table 2:** Summary of best treatment responses

Response, *n* (%)	Observational study		Phase 2 trial		
	All patients (*N* = 133)	Patients not entering phase 2 trial (*n* = 71)	Len (*n* = 31)	Len+Dex (*n* = 31)	Total population (*N* = 62)
OR	105 (79)	43 (61)	31 (100)	31 (100)	62 (100)
≥VGPR	55 (41)	15 (21)	17 (55)	23 (74)	40 (65)
CR	14 (11)	2 (3)	5 (16)	7 (23)	12 (19)
VGPR	41 (31)	13 (18)	12 (39)	16 (52)	28 (45)
PR	50 (38)	28 (39)	14 (45)	8 (26)	22 (35)
MR/SD	19 (14)	19 (27)			
PD	4 (3)	4 (6)			
NE	5 (4)	5 (7)			

OR indicates overall response; CR, complete response; MR, minimal response; NE, not evaluable; PD, progressive disease; PR, partial response; SD, stable disease; VGPR, very good partial response.

**Figure 1:**
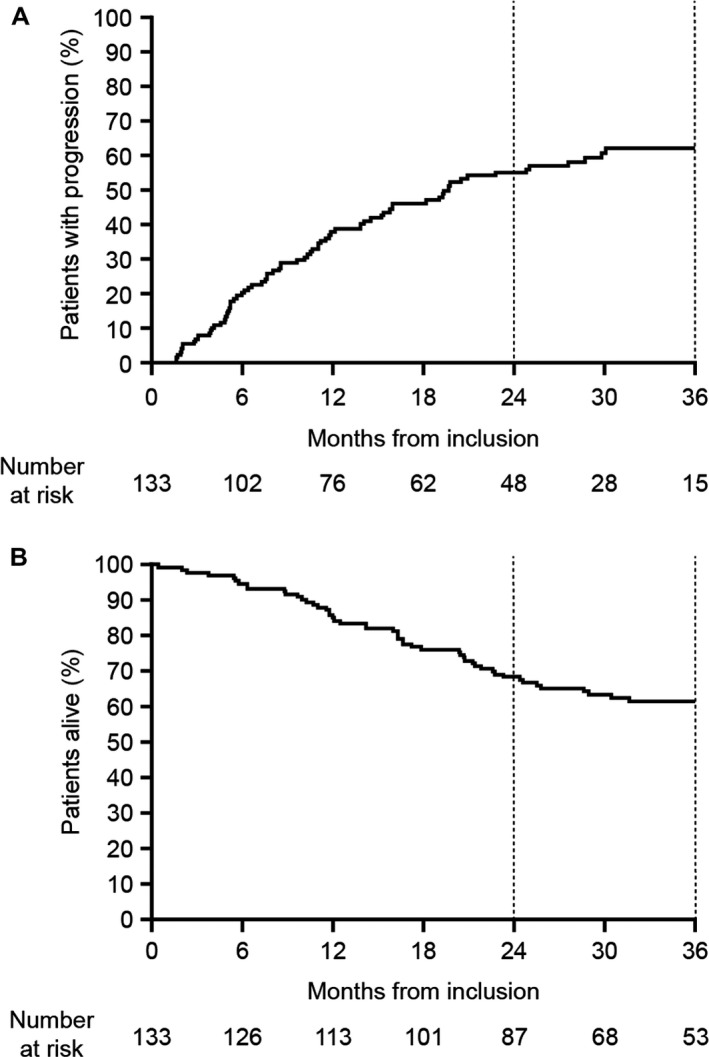
Outcomes in the total observational study population (*N* = 133). (A) TTP measured from the time of inclusion in the study at first relapse. (B) OS measured from the time of inclusion in the study.

After 3.3 years median follow‐up, median TTP in the total observational study population was 19.7 months (95% confidence interval [CI] 14.0–29.0; Fig. 1A). Twenty‐eight percent (95% CI 20–37%) of patients were still on the original study medication at 3 years. PFS and OS rates at this time were 35% (95% CI 26–43%) and 61% (95% CI 52–69%; Fig. 1B), respectively, with disease progression being observed in 62% of patients. In the subgroup of patients who achieved ≥PR in the observational study but did not enter the phase 2 trial (*n* = 43), median TTP was 13.9 months (95% CI 8.6–19.1) after 2.2 years’ median follow‐up; 3‐year PFS and OS were 19% (95% CI 8–34%) and 56% (95% CI 40–69%), respectively. The median DoR and response rate in this subpopulation were 9 months and 19%, respectively.

##### Phase 2 trial

**Figure 2:**
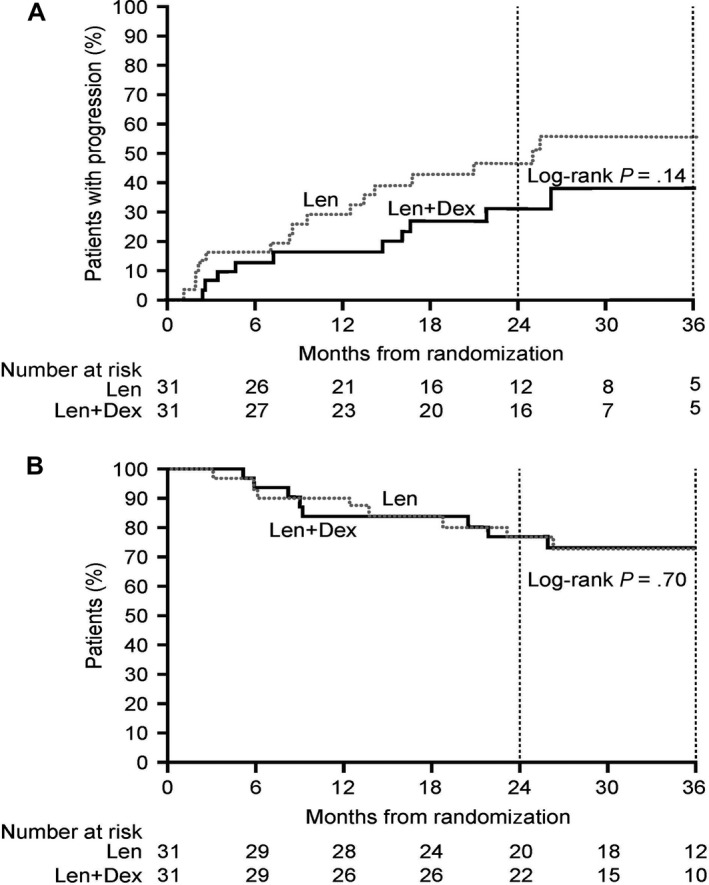
Outcomes in patients treated with Len (*n* = 31) versus Len+Dex (*n* = 31) in the phase 2 clinical trial. (A) TTP measured from randomization. Disease progression in the Len versus the Len+Dex arm was 47% (31–66%) versus 31% (17–52%) at 2 years, and 55% (38–74%) versus 38% (22–61%) at 3 years (*P* = 0.14). (B) OS measured from randomization. Three‐year OS was 73% in both treatment arms (95% CI 53–86% for Len+Dex, 53–85% for Len; *P* = 0.70).

After 26 months’ median follow‐up, median TTP was 24.9 months (12.5–not calculable) versus not reached with Len versus Len+Dex (Fig. 2A). Median TTP was not reached in the total phase 2 trial population (*N* = 62). Response rates at the time of randomization in the phase 2 trial were as follows: CR, 2% (*n* = 1); VGPR, 10% (*n* = 10); and PR, 89% (*n* = 55). Responses deepened with continued treatment in 60% of phase 2 trial participants. Of the 55 patients with a PR at randomization, 26 subsequently achieved a VGPR and seven achieved a CR, while response deepened from an initial VGPR to a CR in four patients. Sixty‐five percent of phase 2 trial participants achieved ≥VGPR as their best response (rates among patients randomized to Len versus Len+Dex were 55% vs. 74%; *P* = not significant), including 19% CRs (16% vs. 23% randomized to Len vs. Len+Dex; *P* = not significant). The median DoR among all phase 2 trial participants was 26.4 months (range 5–57 months).

Three‐year PFS and OS among phase 2 trial participants were 52% (95% CI 38–65%) and 73% (95% CI 60–83%), respectively—significantly higher than the corresponding values reported above for those patients who achieved ≥PR in the observational study but did not enter the phase 2 trial (19% and 56%, respectively; *P* < 0.001 and *P* = 0.04). Three‐year OS was 73% in both treatment arms (Fig. 2B). At the median follow‐up time, the OS in the Len/Dex CR 85%, VGPR 81%, PR 50%, and PFS was CR 71%, VGPR 57%, PR 49%, for the Len group; Os Len CR 82%, VGPR 80%, PR 46%, PFS CR 57%, VGPR 40%, PR 36%.

The impact of cytogenetic abnormalities on progression and OS is shown in Table 3. In the populations of both the observational study and the phase 2 trial, OS and the percentage of progression‐free patients at 3 years were higher among patients with standard‐ versus high‐risk profiles. Among the observational study population, all the investigated mutations except del(17p) significantly impacted the percentage of patients without progression, even with 60% threshold. By contrast, only del(13q) and t(4;14) significantly impacted OS. The OS of the treatment on subsequent relapse from both studies is summarized in Table 4.

**Table 3:** Cytogenetic abnormalities detected by FISH

	*n* (%)	Patients with progression (%)		Overall survival (%)	
		At 2 years	At 3 years (*P*)^a^	At 2 years	At 3 years (*P*)^a^
Observational study					
+1q21	28 (35)	75	79	68	55
No +1q21	52 (65)	44	53 (*0.006*)	77	73 (*0.253*)
del(13q)	25 (31)	80	80	56	48
No del(13q)	56 (69)	41	52 (*0.005)*	84	78 (*0.008*)
del(17p)	8 (10)	75	–	75	50
No del(17p)	73 (90)	50	57 (*0.09*)	75	71 (*0.12*)
t(4;14)	7 (15)	0 (*median=8 months*)	0 (*median=5 months*)	14	0 (*median=21 months*)
No t(4;14)	41 (85)	45	55 (*0.0001*)	83	75 (*<0.001*)
High risk	37 (45)	73	81	67	55
Standard risk	45 (55)	38	46 (*0.001*)	80	78 (*0.07*)
Phase 2 trial					
High risk	20 (50)	57	70	75	65
Standard risk	20 (50)	15	25 (*0.009*)	90	90 (*0.14*)

*P*‐values are for the comparison of patients with versus without the specified abnormality or risk factor.

**Table 4:** The OS of the treatment on subsequent relapse

	*n* (%)	Median, years	At 2, % (*P*)^a^
Bortezomib‐based	43 (57)	2.0	50 (*NS*)
Thalidomide‐based	6 (8)	1.4	0 (*NS*)
Lenalidomide‐based	14 (19)	NR	58 (*NS*)
Other	12 (16)	2.4	50 (*NS*)

a *P*‐values are for the comparison of patients with versus without bortezomib treatment.

### EORTC quality of life questionnaires

**Figure 3:**
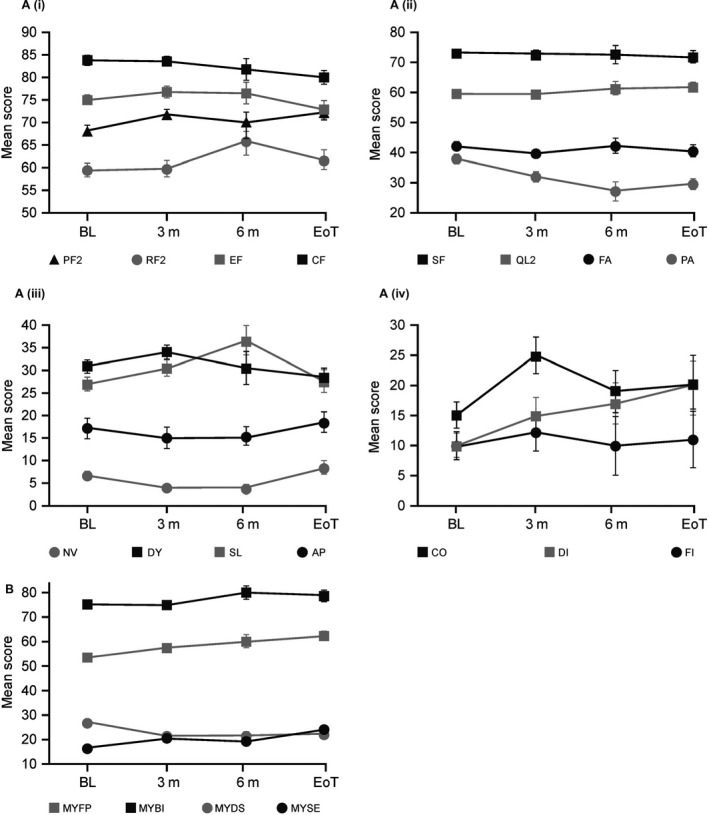
Results from the EORTC quality of life questionnaires, presented as mean and 95% confidence intervals. (A) Results from the core module, EORTC QLQ‐C30. (i) physical functioning (PF2), role functioning (RF2), emotional functioning (EF), cognitive functioning (CF); (ii) social functioning (SF), global health (QL2), fatigue (FA), pain (PA); (iii) nausea and vomiting (NV), dyspnoea (DY), insomnia (SL), appetite loss (AP); (iv) constipation (CO), diarrhea (DI), and financial difficulties (FI). (B) Results from the multiple myeloma module, EORTC QLQ‐MY20: future perspective (MYFP), body image (MYBI), disease symptoms (MYDS), and side effects of treatment (MYSE). BL indicates baseline (*n* = 105); 3 m, at 3 months of treatment (*n* = 61); 6 m, at 6 months of treatment (*n* = 22); EoT, end of trial (1 month after the ninth cycle; *n* = 38).

Figure 3 summarizes the findings from the EORTC QLQ‐C30 and EORTC QLQ‐MY20 questionnaires, which were completed by observational study participants. No significant differences were evident in any of the subscales over time. Insomnia increased during treatment (probably an effect of Dex) but declined to baseline at the end of treatment. Diarrhea also increased during treatment but failed to return to baseline by the end of the study, indicating an increased time to normalize versus insomnia. Future perspective (MYFP) scores increased slightly during treatment and at the end of the study.

### Safety

Table 5 summarizes treatment‐emergent AEs (TEAEs). For phase 2 trial participants, TEAEs that occurred prior to randomization are included within the observational study data. The most common hematologic TEAEs during the observational study were thrombocytopenia (38%), anemia (30%), and neutropenia (13%). Febrile neutropenia was reported in only 2% of the observational study population. Upper respiratory tract infection was the most common nonhematologic TEAE (15%). Thromboembolic events occurred in seven patients (5%).

**Table 5:** Incidence and severity of TEAEs in the overall study populations and in the two arms of the phase 2 trial. All TEAEs are presented

Event	Observational study (*N* = 133)		Phase 2 trial					
			Len (*n* = 31)		Len+Dex (*n* = 31)		Total population (*N* = 62)	
	All grades, *n* (%)	Grade 3–4, *n* (%)	All grades, *n* (%)	Grade 3–4, *n* (%)	All grades, *n* (%)	Grade 3–4, *n* (%)	All grades, *n* (%)	Grade 3–4, *n* (%)
Fatigue	13 (10)	0 (0)	6 (19)	0 (0)	6 (19)	0 (0)	12 (19)	0 (0)
Nausea	3 (2)	0 (0)	3 (10)	0 (0)	3 (10)	0 (0)	6 (10)	0 (0)
Anemia	40 (30)	5 (4)	9 (29)	0 (0)	6 (19)	0 (0)	15 (24)	0 (0)
Thrombocytopenia	50 (38)	7 (5)	20 (65)	1 (3)	16 (52)	1 (3)	36 (58)	2 (3)
Neutropenia	17 (13)	4 (3)	29 (94)	20 (65)	26 (84)	9 (29)	55 (89)	29 (47)
Febrile neutropenia	2 (2)	1 (1)	0 (0)	0 (0)	1 (3)	1 (3)	1 (2)	1 (2)
Pneumonia	15 (11)	9 (7)	1 (3)	0 (0)	5 (16)	3 (10)	6 (10)	3 (5)
Herpes zoster infection^a^	6 (5)	1 (1)	0 (0)	0 (0)	2 (6)	1 (3)	2 (3)	1 (2)
Upper respiratory tract infection	20 (15)	2 (2)	10 (32)	0 (0)	8 (26)	0 (0)	18 (29)	0 (0)
Cough	4 (3)	0 (0)	2 (6)	0 (0)	1 (3)	0 (0)	3 (5)	0 (0)
Urinary tract infection	5 (4)	1 (1)	1 (3)	0 (0)	2 (6)	0 (0)	3 (5)	0 (0)
Diarrhea	4 (3)	0 (0)	9 (29)	0 (0)	8 (26)	1 (3)	17 (27)	1 (2)
Constipation	9 (7)	1 (1)	3 (10)	0 (0)	1 (3)	1 (3)	4 (6)	1 (2)
Deep vein thrombosis^b^	3 (2)	0 (0)	2 (6)	1 (3)	1 (3)	1 (3)	3 (5)	2 (3)
Pulmonary embolism^b^	4 (3)	3 (2)	0 (0)	0 (0)	0 (0)	0 (0)	0 (0)	0 (0)
Back pain	3 (2)	1 (1)	7 (23)	0 (0)	3 (10)	0 (0)	10 (16)	0 (0)
Cataract	0 (0)	0 (0)	2 (6)	0 (0)	2 (6)	1 (3)	3 (5)	1 (2)

a No prophylaxis against herpes zoster virus was used.

b All participants were given prophylaxis with LMWH or ASA according to standard procedures at each clinic.

The most common hematologic TEAE during the phase 2 trial was neutropenia, which occurred in 89% of patients. However, the incidence of febrile neutropenia remained low (2%). Diarrhea was more common in the phase 2 trial than in the observational study, suggesting that this AE may increase with prolonged Len administration. Four patients developed cataracts during this trial—two in each treatment arm. Pneumonia was more common with Len+Dex than with Len; however, TEAE rates overall were similar in the two treatment arms.

#### Serious adverse events

In total, 81 SAEs were reported across the two studies. The most common SAE was pneumonia, occurring in 15 patients; 11 of these cases were recorded during the observational study. Overall, nine‐second primary malignancies (SPMs) were reported in nine patients (7%), although only two occurred during the studies: one anal adenocarcinoma, which occurred in a patient in the Len+Dex arm in the phase 2 trial and was confirmed after 14 months of treatment; and one non‐small‐cell lung carcinoma, which developed within 2 months of treatment in an observational study participant. Two of the nine SPMs were hematologic, both occurring in the phase 2 trial: one large B‐cell lymphoma in a Len+Dex recipient and one acute myeloid leukemia in a single‐agent Len recipient. Both patients had received high‐dose melphalan during first‐line treatment. The remaining SPMs were as follows: pancreatic cancer (*n* = 1); lung cancer (*n* = 1); adenocarcinoma of the bowel (*n* = 2); and basal cell skin cancer (*n* = 1). Four deaths (3%) occurred during the observational study (Fig. S1): one owing to progressive disease; one owing to lung cancer; and two as a result of infectious disease complications (pneumonia and sepsis of unknown origin). One patient (2%) died during the phase 2 trial (cause “sudden death”; no autopsy was performed).

### Flow cytometry characterization of peripheral blood mononuclear cells (phase 2 trial)

To investigate the impact of Len versus Len+Dex on T and NK cells, we carried out a comparative analysis of these two cell types. PBMC samples from 11 and nine Len and Len+Dex recipients, respectively, were collected at randomization and at random timepoints during treatment. Neither the percentage of NK cells nor the expression levels of activating receptors and inhibitory receptors differed significantly either between patients treated with Len and Len+Dex or during treatment versus at trial inclusion (Fig. S2). Importantly, we observed no differences in cell surface expression levels of CD16 or NKG2D molecules, which have previously been shown to be positively impacted by treatment with Len, as previously described^16^.

Further to the above, analyses of T cells and their subsets (memory and naïve), as well as expression levels of costimulation, exhaustion, and energy‐related proteins, indicated no significant differences between patients treated with Len and Len+Dex (Fig. S3). Finally, molecules such as CD16, NKG2D, and CD28, which are essential for the control and elimination of myeloma, were highly expressed in all the patient material analyzed, indicating that neither Len nor Len+Dex prolonged treatment at first relapse reduces either the expression or, potentially, the activity of these cells 16, 17, 18, 19.

## Discussion

In the two studies reported here, OS did not differ in patients with RRMM who received Len versus Len+Dex as maintenance therapy following a response to initial Len+Dex treatment at first relapse. Although there was an apparent trend toward increased TTP in the Len+Dex arm, this did not reach statistical significance. Additionally, we observed no significant differences in the phenotype of circulating lymphocytes including cytotoxic T cells, helper T cells, and NK cells among Len versus Len+Dex recipients, and previous studies demonstrated that concomitant treatment with either low‐ (160 mg/cycle) or high‐dose (480 mg/cycle) Dex may result in inhibition of the immunomodulatory effects of Len 20, 21. In these studies, Dex treatment also reduced the expression of activating receptors such as NKG2D in NK cells. In contrast, we observed no changes in the levels of expression of these activating molecules following treatment with either Len or Len+Dex in our phase 2 clinical trial. One potential explanation for these differing results could be that all the patients in our phase 2 trial had already responded to Len+Dex in the preceding observational study, and therefore were able to tolerate the immunosuppressive effects of Dex. Our results do not exclude the possibility that Len+Dex may prolong TTP versus Len alone, but this is unconfirmed and must be balanced against the potential increased risk of AEs with Len+Dex.

In Europe, Len plus low‐dose Dex is the standard treatment for MM at first relapse; it is also becoming established as a standard therapeutic option for newly diagnosed MM in patients who are ineligible for ASCT. As all the participants in our observational study had received only one prior line of treatment, the results obtained confirm the efficacy of Len+Dex as a first‐line treatment for RRMM.

Our results are broadly consistent with those of previous studies, although differing study methods and patient populations confound direct comparison. In transplant‐ineligible patients with newly diagnosed MM in the FIRST trial, median PFS was prolonged with Len+Dex maintenance administered continuously until disease progression (25.5 months) versus Len+Dex administered for a fixed period of 18 months (20.7 months) or melphalan–prednisone–thalidomide administered for 18 months (21.2 months) 22. The 3‐year PFS rate with continued Len+Dex was ~40% 22. In our phase 2 trial, median PFS had not been reached at 3 years in patients who received prolonged therapy with Len+Dex following a response to initial Len+Dex treatment, and the PFS rate was 60%. Corresponding values in the subgroup of patients who responded to initial treatment but did not enter the phase 2 study were 13.9 months and 19%. In two randomized trials that included continuous Len+Dex as a control arm (the ASPIRE study 23 [carfilzomib plus Len+Dex vs. Len+Dex] and TOURMALINE‐MM1 24 [ixazomib plus Len+Dex vs. Len+Dex] trials, both performed in patients with MM who had received 1–3 prior therapies), median PFS with Len+Dex was 17.6 months and 14.7 months, respectively. Our data suggest that continuous Len+Dex improves outcome vs. fixed‐duration therapy, but limitations in the studies prevent us from drawing this conclusion.

Another major determinant of treatment outcome is the presence or absence of high‐risk cytogenetic aberrations. Rates of disease progression (based on TTP, rather than PFS, data) in our observational study were nearly doubled at 3 years in patients with versus those without high‐risk cytogenetic features and were increased nearly threefold in the phase 2 trial, while 3‐year OS rates in patients with high‐risk cytogenetics were reduced by nearly 30% versus standard‐risk patients in both studies. Forty‐five percent and 50% of patients in the observational study and the phase 2 trial, respectively, had high‐risk cytogenetic features.

Regarding safety, the hematologic TEAEs neutropenia and thrombocytopenia were more common with prolonged (phase 2 trial) versus short‐term (observational study) Len+Dex treatment. However, these events were managed successfully: Neutropenia was treated with granulocyte colony‐stimulating factor, while thrombocytopenia was managed with occasional discontinuation of anticoagulants. Rates of febrile neutropenia were similar in the two studies. The overall pattern of AEs in our studies was consistent with that observed in previous studies, in which neutropenia was also reported as the most common grade 3–4 AE with either Len or Len+Dex 6, 8, 9, 10. The longer duration of treatment in the phase 2 trial had little impact on the rates of grade 3–4 AEs, with the exception of neutropenia. Comparison of TEAEs in the two arms of the phase 2 trial suggests that Len and Len+Dex were similarly well tolerated. However, patient numbers were relatively small, and it remains possible that treatment with Len+Dex could place patients at increased overall risk of TEAEs. With prolonged treatment, one should also consider the risk of second primary malignancies (SPM) associated with Len. We observed a total of nine SPMs (7%) during the study and the follow‐up time. This risk must always be compared with the putative benefits from the drug.

Our studies had some limitations. As the first study was observational, the data were not fully comprehensive—for example, β_2_‐microglobulin levels were not available. There was no lower limit for CrCl; however, only two patients had CrCl <30 mL/min at baseline, and no patient had CrCl <15 mL/min. Therefore, the applicability of our results to patients with renal impairment is uncertain. As the phase 2 trial was initiated in 2010, measurement of minimal residual disease was not included in the protocol. The majority of patients had ISS stage I or II disease, making it impossible to stratify the results by ISS stage.

In conclusion, our results indicate that long‐term (up to 24 months) prolonged treatment with Len with or without Dex provides sustained, clinically relevant responses. The longer duration of treatment in the phase 2 trial was associated with increased, but manageable, AEs beside the SPMs. After achievement of a response to initial Len+Dex treatment, OS did not differ between patients subsequently treated with Len and Len+Dex at either 2 or 3 years. There was a trend toward prolonged TTP with Len+Dex versus Len, but this did not reach statistical significance and, when selecting the most appropriate treatment, possible benefits must be balanced against the potential risk of AEs. There was a clear trend toward increased depth of response and PFS with Len‐Dex over Len. A larger study may have met statistical significance.

## Supporting information


**Figure S1.** Patient disposition: A. phase 4 study and B. phase II study.
**Figure S2.** Flow cytometry analyses of NK cells.
**Figure S3.** Flow cytometry analyses of T cells.
