# Supplementary material for: Lenalidomide versus lenalidomide + dexamethasone prolonged treatment after second‐line lenalidomide + dexamethasone induction in multiple myeloma
Source: Cancer Med. 2018 Apr 19;7(6):2256–68. doi: 10.1002/cam4.1422 (PMC6010717; doi:10.1002/cam4.1422)
Supplement: Supplementary file 1 — Figure S1. Patient disposition: A. phase 4 study and B. phase II study. Figure S2. Flow cytometry analyses of NK cells. Figure S3. Flow cytometry analyses of T cells. [file CAM4-7-2256-s001.pdf]

SUPPLEMENTAL FIGURES

Supplemental figure S1. Patient disposition: A. phase IV study and B. phase II study

A.

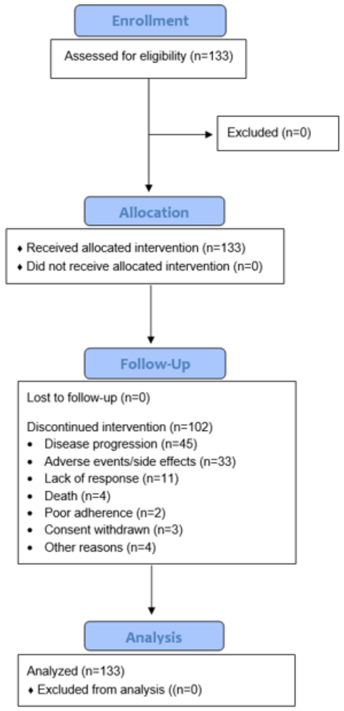

B.

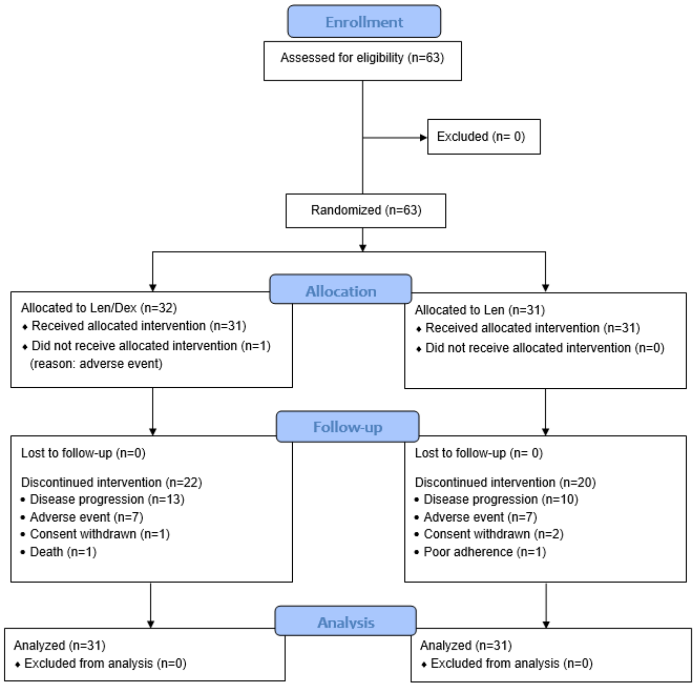

Supplemental figure S2. Flow cytometry analyses of NK cells.

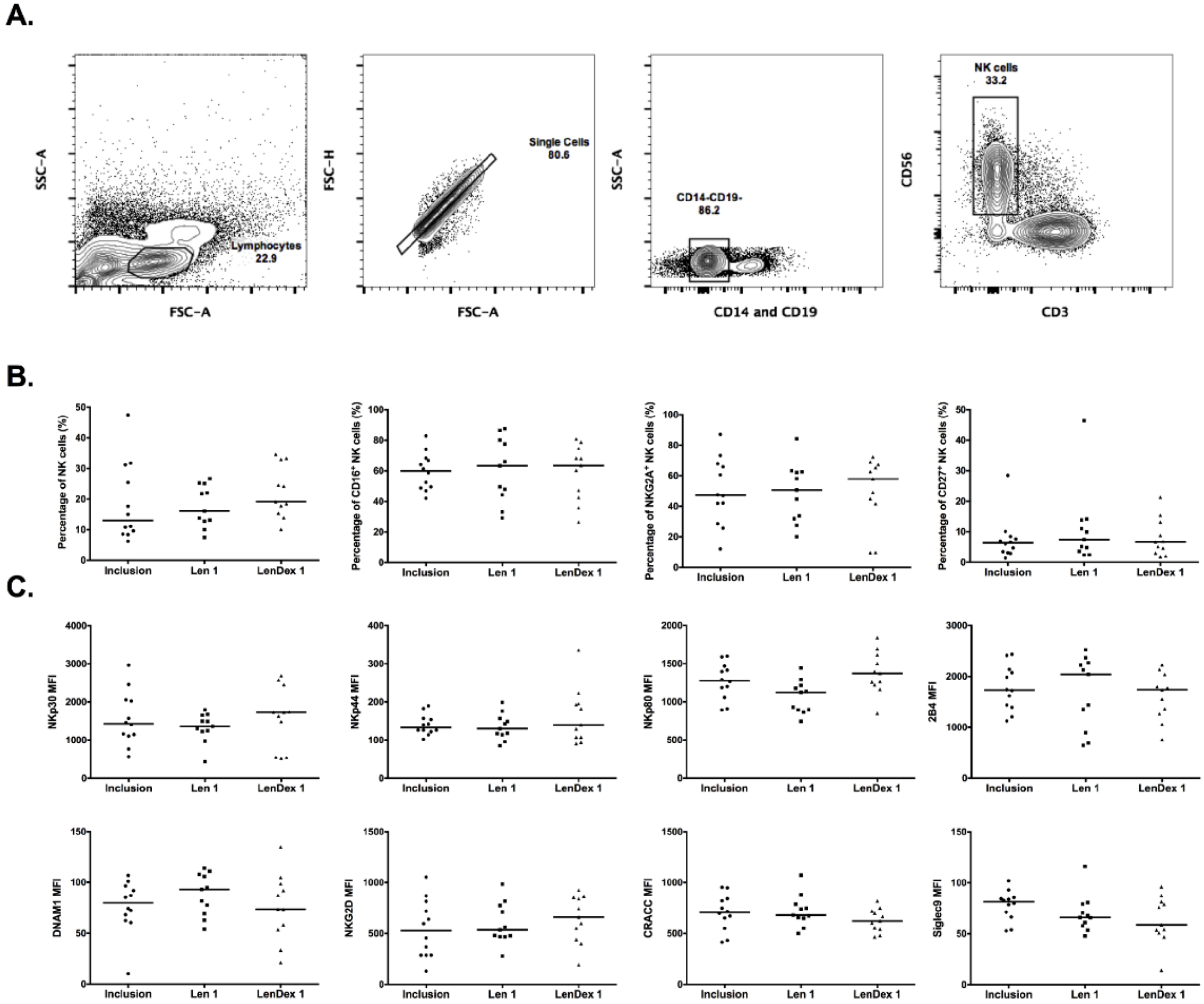

FSC, forward scatter; MFI, mean fluorescence intensity; SSC, side scatter.

Supplemental figure S3. Flow cytometry analyses of T cells.

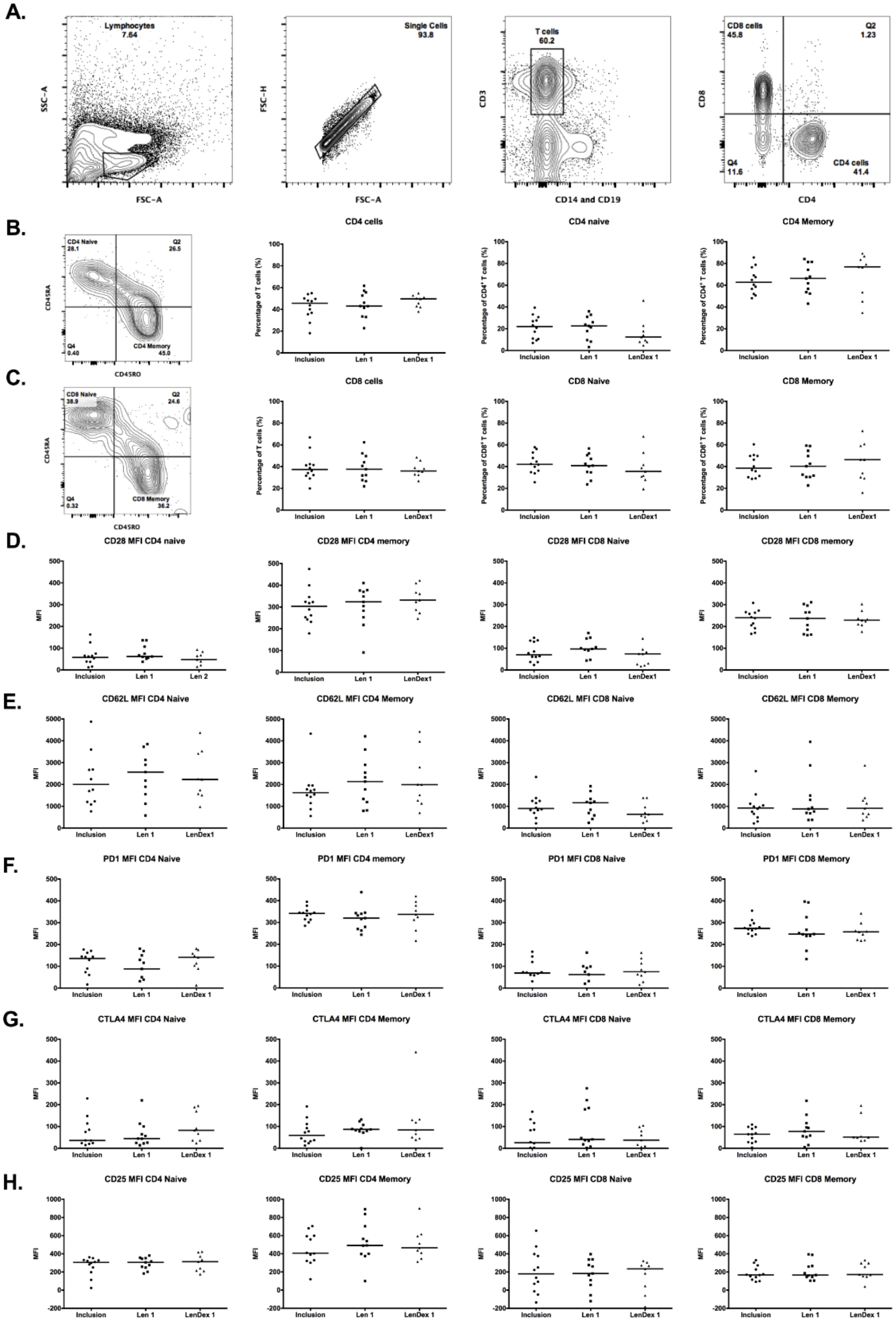

FSC, forward scatter; MFI, mean fluorescence intensity; SSC, side scatter.
